# Supporting women’s health outcomes after breast cancer treatment comparing a text message intervention to usual care: the EMPOWER-SMS randomised clinical trial

**DOI:** 10.1007/s11764-022-01209-9

**Published:** 2022-04-23

**Authors:** Anna C. Singleton, Rebecca Raeside, Stephanie R. Partridge, Karice K. Hyun, Justin Tat-Ko, Stephanie Che Mun Sum, Molly Hayes, Clara K. Chow, Aravinda Thiagalingam, Katherine Maka, Kerry A. Sherman, Elisabeth Elder, Julie Redfern

**Affiliations:** 1 Engagement and Co-design Research Hub, School of Health Sciences, Faculty of Medicine and Health, The University of Sydney, Sydney, NSW Australia; 2 Prevention Research Collaboration, Charles Perkins Centre, The University of Sydney, Sydney, NSW Australia; 3 Department of Cardiology, Concord Repatriation General Hospital, Sydney, NSW Australia; 4 Westmead Applied Research Centre, Faculty of Medicine and Health, The University of Sydney, Sydney, NSW Australia; 5 Department of Cardiology, Westmead Hospital, Sydney, NSW Australia; 6 George Institute for Global Health, University of New South Wales, Sydney, NSW Australia; 7 Research Education Network, Western Sydney Local Health District, Sydney, NSW Australia; 8 Department of Physiotherapy, Westmead Hospital, Sydney, NSW Australia; 9 Westmead Breast Cancer Institute, Westmead Hospital, Sydney, NSW Australia; 10 Centre for Emotional Health, Department of Psychology, Macquarie University, Sydney, NSW Australia

**Keywords:** Text messaging, Breast cancer, Cancer survivorship, Mobile health, Randomised controlled trial, Telemedicine

## Abstract

**Purpose:**

The aim of this study is to evaluate the efficacy, feasibility and acceptability of a co-designed lifestyle-focused text message intervention (EMPOWER-SMS) for breast cancer survivors’ self-efficacy, quality of life (QOL), mental (anxiety, depression, stress) and physical (endocrine therapy medication adherence, physical activity, BMI) health.

**Methods:**

Single-blind randomised controlled trial (1:1) comparing EMPOWER-SMS to usual care at 6-months (intention-to-treat). Setting: public Breast Cancer Institute (Sydney, Australia). Eligibility criteria: adult (> 18 years) females, < 18-months post-active breast cancer treatment (stage I-III), owned a mobile phone, written informed consent. Primary outcome: Self-Efficacy for Managing Chronic Disease Scale at 6 months. Process data: message delivery analytics, cost, and post-intervention survey.

**Results:**

Participants (*N* = 160; mean age ± SD 55.1 ± 11.1 years) were recruited 29th-March-2019 to 7th-May-2020 and randomised (*n* = 80 EMPOWER-SMS: *n* = 80 control). Baseline mean self-efficacy was high (I: 7.1 [95%CI 6.6, 7.5], C: 7.4 [7, 7.8]). Six-month follow-up: no significant differences between groups for self-efficacy (I: 7.6 [7.3, 7.9], C: 7.6 [7.3, 7.9], adjusted mean difference 0 (95%CI 0.4, 0.4), QOL, mental health, physical activity, or BMI. Significantly less EMPOWER-SMS participants missed ≥ 1 endocrine therapy medication doses compared to control (I: 3/42[7.1%], C: 8/47[17.0%], Adjusted RR 0.13 [95%CI 0.02, 0.91]). Text messages were delivered successfully (7925/8061, 98.3%), costing $13.62USD/participant. Participants *strongly/agreed* EMPOWER-SMS was easy-to-understand (64/64; 100%), useful (58/64; 90.6%), motivating for lifestyle change (43/64; 67.2%) and medication adherence (22/46; 47.8%).

**Conclusion:**

EMPOWER-SMS was feasible, inexpensive, acceptable for delivering health information to breast cancer survivors between medical appointments, with minor improvements in medication adherence.

**Implications for Cancer Survivors:**

Text messages offer a feasible strategy for continuity-of-care between medical appointments.

**Supplementary Information:**

The online version contains supplementary material available at 10.1007/s11764-022-01209-9.

## Introduction

Breast cancer is the most commonly diagnosed cancer among women worldwide and five-year survival rates are high (85–90% in high-income countries) [1]. However, the number of lost disability adjusted life years [DALYs] among breast cancer survivors is high globally (17,708,600 DALYs) [1]. The greatest contributors to DALYs are modifiable risk factors, including overweight and obesity, unhealthy diet, physical inactivity and smoking [1]. Managing modifiable risks can be challenging due to treatment and endocrine therapy medication side effects (e.g. hot flushes, fatigue) that negatively affect women’s mental and physical health [2]. Health education can improve self-efficacy (self-confidence) for managing health [3–6] and promote healthy lifestyles after active treatment (surgery, chemotherapy, radiation) [7]. However, survivorship education programs are scarce [8], resource-intensive, and rarely co-designed with end-users, which may reduce effectiveness [7]. Novel, scalable and co-designed post-treatment health programs are urgently needed.

Mobile health (mHealth) interventions (e.g. mobile applications, text messaging) are strategies for providing health information remotely [9]. The most accessible mHealth intervention is text messages, as over 5 billion people own mobile phones globally [10]. Randomised clinical trials (RCTs) show that health education via text messages is effective for improving various modifiable risks [11–14]. There is limited but growing evidence that text message interventions may improve modifiable risks for individuals with breast cancer, including adherence to endocrine therapy medication [12] and weight maintenance [13]. However, few interventions were co-designed with breast cancer survivors [15].

Our team co-designed the EMPOWER-SMS text message program with breast cancer survivors, health professionals and researchers, which aims to improve breast cancer survivors’ health self-efficacy, quality of life (QOL), mental (anxiety, depression, stress) and physical (BMI, physical activity, medication adherence) health [16]. We hypothesized that participants who received the six-month EMPOWER-SMS intervention would have improved self-efficacy, QOL and health outcomes compared to usual care (control group) at six-month follow-up.

## Methods

### Trial design

The study protocol is published [17]. Briefly, this study was a single-blind RCT (1:1) comparing EMPOWER-SMS to usual care for improving breast cancer survivors’ self-efficacy, QOL and mental and physical health at 6-month follow-up. The study followed the Consolidated Standards of Reporting Trials (CONSORT) guidelines (Supplementary Material 1: CONSORT checklist). Ethics approval was received from Western Sydney Local Health District Human Ethics Research Committee (AU RED HREC/18/WMEAD/281) and all participants provided written informed consent. Clinical trial registration: ANZCTR12618002020268, http://www.who.int/trialsearch/Trial2.aspx?TrialID=ACTRN12618002020268, 17-December-2018). University of Sydney was the study sponsor.

### Study population

Participants were recruited from the Westmead Breast Cancer Institute in Western Sydney, Australia that serves a culturally and socioeconomically diverse population. Inclusion criteria: adult (> 18 years) females, diagnosed with early-stage (0–III) breast cancer, within 18 months of finishing active breast cancer treatment (could be taking endocrine therapy medication, e.g. Tamoxifen, Aromatase inhibitors) and owned a mobile phone. Exclusion criteria: diagnosed with metastatic breast cancer or insufficient English to provide consent.

### Control group

The control group received usual medical care according to the breast cancer institute, which included access to breast care nursing and allied health support (psychologist, dietician, physiotherapist). Participants received one ‘welcome’ text message at baseline, containing their group allocation, and one ‘follow-up interview reminder’ text message at six-months.

### Intervention group

EMPOWER-SMS aimed to provide supportive health education and was co-designed by breast cancer survivors, health professionals and researchers [16]. Participants received usual care, a ‘welcome’ text message, EMPOWER-SMS and a ‘follow-up interview reminder’ message. EMPOWER-SMS delivered four text messages per week for 6 months (104 messages total) regarding (i) physical activity and healthy diet, (ii) social and emotional wellbeing, (iii) medication adherence and side effects management and (iv) general breast cancer information. Participants taking endocrine therapy medications received 26 text messages from each topic. Other participants received messages regarding physical activity and healthy diet (39/104; 37.5%), social and emotional wellbeing (27/104; 26.0%) and general breast cancer information (38/104; 36.5%). Messages were positively toned, semi-personalised with the participant’s preferred name and designed to be appropriate for individuals with a Grade 7 (Flesch Kincaid) reading level [16]. One-third (33/104; 33.0%) of messages included weblinks to additional science-based information and free resources. Participants were advised not to reply (one-way delivery). However, for safety, an unblinded health counsellor monitored the automated text message delivery system for replies.

### Outcomes

Study visits were in-person at baseline and 6 months [17]. Briefly, at baseline, participants provided self-reported demographics (age, sex, ethnicity, country of birth), medical history and chronic disease risk factors (confirmed in medical files). The primary outcome was self-efficacy measured by the Self-Efficacy for Managing Chronic Disease Scale [18] (Cronbach alpha range: 0.88–0.95) [19], which measures confidence for managing six domains (fatigue, physical discomfort, emotional distress, other health problems, achieving health management tasks and things other than medication) on a scale from 1 to 10 (not confident at all to totally confident). The mean score out of 10 across domains is calculated, with higher scores reflecting higher self-efficacy. Secondary outcomes included:

#### Clinical and lifestyle measures

BMI (healthy range: BMI ≤ 25kg/m2), body fat percentage and skeletal muscle mass measured using the Seca medical Body Composition Analyser (Seca GmbH & Co. KG, Hamburg, Germany) [20] and waist circumference (cm) measured using standard tape measurement [21]. Self-reported nutrition behaviours included number of fruits, vegetables, red meat and standard alcoholic drinks consumed in 7 days [22]. Self-reported physical activity was measured using the Global Physical Activity Questionnaire [GPAQ] [21] and validated using an ActiGraph™ GT3X+ (ActiGraph, Pensacola, FL) accelerometer and wear-time log-book with 32/160 (20%) participants [23]. Accelerometers were worn for 7 days after baseline and follow-up visits and then completed the GPAQ. Adherence to endocrine therapy medication was measured by self-reported missed doses within the last 7 days. Australian guideline cut points [24, 25] are presented in Table 2.

#### QOL and mental health

QOL (European Organization for Research and Treatment of Cancer QOL Questionnaire–Core [EORTC QLQ-C30]; and Breast Cancer subscale [EORTC QLQ-BR23]) [26]. Two questions about health and QOL during the past week (7-point Likert scale; 1: very poor to 7: excellent) form the global health status/QOL score that is transformed to a 0–100 scale; higher scores represent higher QOL. Depression Anxiety and Stress scale (DASS-21) measures occurrences of certain behaviours on a 4-point Likert scale (0 = does not apply to me, 3 = Applied to me most of the time) [27]. Depressive, anxiety and stress symptom subscales each have 7-items and scores are doubled (range 0–42); higher scores reflect higher depressive, anxiety and stress symptoms.

### Illness perceptions

Brief Illness Perception Questionnaire [BIPQ] [28] is scored on a 10-point Likert scale across eight domains: disease consequences, timeline, personal control, treatment effectiveness, symptoms, concern, illness understanding, affected emotionally. The final question asks what participants believe were the three most important causes of their breast cancer (free-text response), which were analysed thematically.

### Sample size

A mean difference of 1 (SD 2.05) on the Self-Efficacy for Managing Chronic Disease Scale (6 items) between EMPOWER-SMS and control at 6 months was considered clinically meaningful [18]. A total of 160 participants (80 EMPOWER-SMS: 80 control) were needed to achieve 80% power, with a 5% type I error rate and 20% dropout rate.

### Randomisation and masking

Participants were randomised in a 1:1 (EMPOWER-SMS: control) allocation ratio, using a secured central computer-based randomisation service (R statistical software version 3.6.1; ©The R Foundation). Group allocation was automatically concealed using computer software (Research Electronic Data Capture [REDCap]), which revealed codes ‘Group A’ or ‘Group B’ to the researcher, maintaining researcher blinding. A subsample of 32/160 (20%) participants were randomised to wear an accelerometer, stratified by group (16 EMPOWER-SMS; 16 control), which notified researchers using a computer-generated notification. On the Monday after enrolment, the blinded researcher submitted the ‘Group A’ or ‘Group B’ allocation into the text message software, which automatically sent the ‘welcome’ text message containing participants’ group allocation (EMPOWER-SMS or control). Participants were instructed not to share their group allocation with the research team. A blinded researcher conducted the follow-up interview.

### Statistical methods

Analyses were pre-specified and performed according to the intention-to-treat principle by a blinded statistician [17]. Primary and secondary outcomes were summarised as means and corresponding 95% confidence intervals (CIs) or standard deviations (SD), or if the distribution was skewed, as medians and interquartile intervals (IQI) for continuous variables and as frequencies and percentages for categorical variables. The outcomes were compared between EMPOWER-SMS and control groups at six-months, adjusting for the baseline measure of the outcome, with a significance level of 0.05. Dichotomous outcomes were analysed using log-binomial regression and for continuous outcomes, the analysis of covariance (ANCOVA).

To validate the GPAQ, metabolic equivalents (MET) minutes/day for moderate-to-vigorous physical activity (MVPA) were compared between the self-reported and accelerometer-assessed physical activity. Freedson and colleagues (1998) [23] cut-points were used to define accelerometer MVPA. Total MVPA minutes were divided by the number of days participants wore the accelerometer and MET minutes/day was estimated: minutes of moderate activity × 4 METs plus minutes of vigorous activity × 8 METs. The median MET minutes/day and IQI for accelerometer-assessed and self-reported physical activity were reported, and Spearman correlation coefficients were used to assess the correlation between the measurements. MET minutes/day at follow up was compared between EMPOWER-SMS and control groups using exact Wilcoxon rank sum test.

### Process evaluation

#### Program delivered as planned

Automated text message delivery software (April 2019–November 2020) collected the number of text messages that were sent, delivered successfully or unsuccessfully (‘bounced’), resulted in an ‘opt-out’ or a reply (number, content, and type [text, photo, ‘reaction’, ‘emoji’]). ‘Reactions’ are when a participant clicks on a text message, then clicks that they ‘liked’, ‘loved’ or ‘laughed at’ the message.

#### Program delivery costs

Text message delivery data were used to estimate the cost per person of intervention delivery. Total staff time dedicated to intervention monitoring was estimated.

#### Program acceptability and utility

End-of-study feedback survey data were collected, including 13 questions: ten 5-point Likert-scale items (1 = strongly disagree to 5 = strongly agree) and three yes/no. Question topics included participants’ perceived acceptability and usefulness of repeated text messages, delivery timing, and content suitability for breast cancer survivors.

## Results

### Characteristics of the participants

**Fig. 1:**
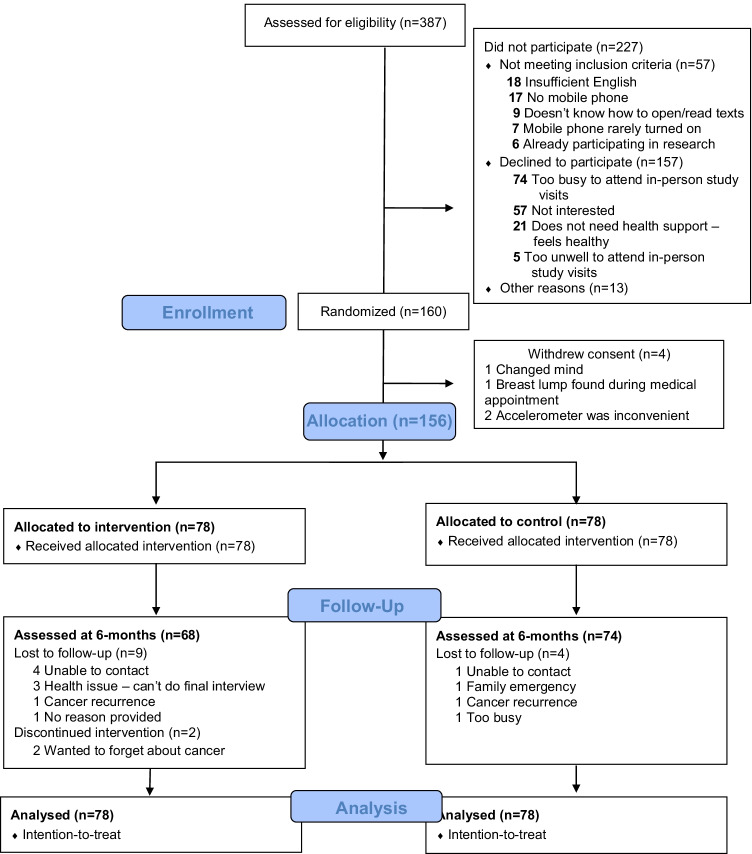
CONSORT flow diagram

From March 2019 to May 2020, 387 patients were assessed for eligibility and approached; 227 did not participate (157 declined; 57 did not meet inclusion criteria) and 160 enrolled and were randomised (see Fig. 1). Due to COVID-19 restrictions, 4/160 (2.5%) participants were recruited and 79/160 (49%) completed follow-up visits over the phone and provided self-reported weight, waist circumference and height measurements based on detailed instructions from the research team. Before completing the baseline visit or receiving the text message with the group allocation, four participants withdrew consent (reasons provided in Fig. 1). Therefore, 156 were allocated to EMPOWER-SMS (*n* = 78) or control (*n* = 78) groups and were included in the analysis. At 6 months, 14/156 (9.0%) participants did not complete the follow-up visit and 2/78 (2.5%) discontinued the intervention (Fig. 1).

At baseline, participants were mean ± SD of 8.0 ± 5.0 months post-active treatment, mean age ± SD 55.0 ± 11.0 years and characteristics were not significantly different between groups, except the EMPOWER-SMS group had more smokers and ex-smokers compared to control (Table 1). No significant differences in primary or secondary outcomes between groups were found at baseline, except EMPOWER-SMS group had higher mean vegetable intake, and higher proportion of people who consumed > 1 standard alcoholic drink per week compared to the control group (Table 2). Overall, few participants met post-treatment secondary prevention guidelines at baseline or were within healthy ranges for depression, anxiety or stress, and significantly fewer EMPOWER-SMS participants met guidelines for waist circumference than controls (Supplementary Material 2). Baseline QOL subscales revealed EMPOWER-SMS participants had significantly lower scores for body image and higher sexual enjoyment than controls (Supplementary Material 3).

**Table 1:** Baseline characteristics

Characteristics	No./Total (%)		
	EMPOWER-SMS (*n* = 78)	Control (*n* = 78)	Total (*N* = 156)
Time between finishing active treatment to enrolment, mean months (SD^+^)	8 (5)	8.1 (5)	8 (5)
Demographics			
Age (years), mean (SD)	53.8 (9.6)	55.7 (12.1)	54.8 (10.9)
Ethnicity			
Caucasian	37/78 (47.4)	32/78 (41.0)	69/156 (44.2)
South Asian (Bangladesh, India, Nepal, Pakistan, Sri Lanka)	8/78 (9.0)	13/78 (16.7)	21/156 (13.4)
Other Asian	10/78 (12.8)	20/78 (25.6)	56/156 (35.9)
Other	18/78 (23.1)	13/78 (16.7)	31/156 (19.9)
Country or region of birth			
Australia	37/78 (47.4)	27/78 (34.6)	64/156 (41)
Europe/United Kingdom	6/78 (7.8)	8/78 (10.2)	14/78 (17.9)
India	5/78 (6.4)	8/78 (10.2)	13/78 (16.7)
Middle East (Including Pakistan, Afghanistan, Egypt)	3/78 (3.8)	7/78 (9.0)	10/78 (12.8)
Southeast Asia (Philippines, Thailand, Laos)	11/78 (14.1)	10/78 (12.8)	21/78 (26.9)
Pacific Islands (Including New Zealand, Tonga, Fiji)	9/78 (11.5)	6/78 (7.7)	15/78 (19.2)
Other	7/78 (9.0)	12/78 (15.4)	19/156 (24.0)
Education			
Year (Grade) 12 or below	26/76 (34.3)	18/78 (23.1)	44/154 (28.6)
Diploma/technical degree	22/76 (28.9)	21/78 (26.9)	43/154 (27.9)
Undergraduate/postgraduate degree	28/76 (36.8)	39/78 (50.0)	67/154 (43.5)
Marital status			
Single/widowed	16/77 (20.8)	12/78 (15.4)	28/155 (18.1)
DeFacto/married	50/77 (64.9)	55/78 (70.5)	105/155 (67.7)
Separated/divorced	11/77 (14.3)	11/78 (14.1)	22/155 (14.2)
Employment status			
Working full/part time	49/75 (65.3)	44/78 (56.4)	93/153 (60.8)
Unemployed	9/75 (12.0)	13/78 (16.7)	22/153 (14.4)
Retired	14/75 (18.7)	18/78 (23.1)	32/153 (20.9)
Other	3/75 (4)	3/78 (3.8)	6/153 (3.9)
Children, # yes	61/78 (78.2)	68/78 (87.2)	129/156 (82.7)
Medical history			
Tumour removal surgery	78/78 (100)	77/78 (98.7)	155/156 (99.4)
Radiotherapy	69/78 (88.5)	69/78 (88.5)	138/156 (88.5)
Chemotherapy	48/77 (62.3)	50/78 (64.1)	98/155 (63.2)
Endocrine therapy	52/78 (66.7)	55/78 (70.5)	107/156 (68.6)
Targeted therapy	12/78 (15.4)	14/77 (18.2)	26/155 (16.8)
High cholesterol diagnosis	11/78 (14.1)	18/78 (23.1)	29/156 (18.6)
High blood pressure diagnosis	17/78 (21.8)	27/78 (34.6)	44/156 (28.2)
CVD diagnosis	3/78 (3.8)	4/78 (5.1)	7/156 (4.5)
Smoking status			
Current smoker	8/78 (10.3)	4/78 (5.1)*	12/156 (7.7)
Ex-smoker	31/78 (39.7)	15/78 (19.2)*	46/156 (29.5)
Never smoked	39/78 (50)	59/78 (75.6)*	98/156 (62.8)

^**+**^Standard deviation; ^*^p < 0.05

**Table 2:** Primary and secondary outcomes at baseline and 6-month follow-up

	Baseline			Six-month follow-up		
	EMPOWER-SMS (*n* = 78)	Control (*n* = 78)		EMPOWER-SMS (*n* = 78)	Control (*n* = 78)	
				Mean months (SD)	Mean months (SD)	Mean months (SD)
Time from randomisation to follow-up				7.1 (1.6)	7.1 (1.5)	7.1 (1.5)
Primary outcome	Mean (95%CI)	Mean (95%CI)	Mean difference (95%CI)	Adjusted mean (95%CI)	Adjusted mean (95%CI)	Adjusted mean difference (95%CI)
Self-efficacy	7.1 (6.6, 7.5)	7.4 (7, 7.8)	− 0.3 (− 1, 0.3)	7.6 (7.3, 7.9)	7.6 (7.3, 7.9)	0 (− 0.4, 0.4)
Secondary outcomes						
Quality of life	69 (63.8, 74.2)	70.4 (65.3, 75.6)	− 1.4 (− 8.8, 5.9)	74.3 (70.9, 77.7)	73 (69.8, 76.2)	1.3 (− 3.3, 6)
Depressive symptoms	18.8 (15.1, 22.4)	17.6 (14.1, 21.2)	1.1 (− 4, 6.2)	17.8 (14.6, 21.1)	18.5 (15.5, 21.5)	− 0.7 (− 5.1, 3.7)
Anxiety symptoms	20.7 (16.4, 25)	17.8 (13.6, 22)	2.9 (− 3.1, 8.9)	19.3 (15.8, 22.8)	20.1 (16.8, 23.4)	− 0.8 (− 5.6, 4)
Stress symptoms	17 (13.3, 20.7)	14.5 (10.9, 18.2)	2.5 (− 2.8, 7.7)	14.5 (11.5, 17.6)	15.6 (12.7, 18.4)	− 1 (− 5.2, 3.1)
Physical activity, METS^$^	1668.7 (1108.2, 2229.3)	1805.4 (1244.9, 2366)	− 136.7 (− 929.5, 656)	1940 (1344.7, 2535.4)	1747.4 (1152, 2342.8)	192.6 (− 649.5, 1034.8)
Clinical						
BMI^&^ kg/m^2^	33.7 (27.6, 39.9)	27.6 (21.4, 33.7) (*n* = 77)	6.2 (− 2.5, 14.9) (*n* = 155)	29 (27.6, 30.4) (*n* = 60)	27.5 (26.2, 28.9) (*n* = 73)	1.4 (− 0.5, 3.4)
Waist circumference, cm	95.3 (92.2, 98.3)	91.4 (88.3, 94.4) (*n* = 76)	3.9 (− 0.4, 8.2) (*n* = 154)	93.1 (91.7, 94.5) (*n* = 56)	92.1 (90.8, 93.3) (*n* = 68)	1 (− 0.8, 2.9)
Fat Mass Percentage %	42.7 (41.3, 44.1) (*n* = 74)	41.4 (40, 42.9) (*n* = 71)	1.3 (− 0.7, 3.3) (*n* = 145)	41.9 (41, 42.9) (*n* = 25)	42.9 (41.9, 43.9) (*n* = 27)	− 1 (− 2.4, 0.4)
Skeletal muscle mass Percentage %	25.4 (24.8, 25.9) (*n* = 74)	25 (24.3, 25.6) (*n* = 70)	0.4 (− 0.4, 1.3)	24.6 (24.1, 25.1) (*n* = 25)	24.7 (24.2, 25.2) (*n* = 27)	− 0.1 (− 0.8, 0.6)
Lifestyle						
Endocrine medication adherence (≥ 1 missed doses in the last 7 days)	6/46 (13)	6/51 (11.8)	1.28 (− 11.87, 14.43)^	3/42 (7.1)	8/47 (17)	**0.13 (0.02, 0.91)**^+*^
Servings of fruit per day, mean (SD^#^)	1.7 (1.5, 2) (*n* = 77)	1.7 (1.5, 1.9)	0.1 (− 0.3, 0.4)	1.5 (1.3, 1.7)	1.7 (1.5, 1.9)	− 0.2 (− 0.4, 0)
Servings of vegetables per day, mean (SD)	4.5 (4.1, 4.9) (*n* = 77)	3.7 (3.3, 4.1)	0.8 (0.2, 1.4)**	3.9 (3.5, 4.4)	4.2 (3.8, 4.6)	− 0.3 (− 0.9, 0.3)
Servings of red meat per week, mean (SD)	2.6 (2.1, 3) (*n* = 77)	2 (1.6, 2.5)	0.5 (− 0.1, 1.2)	1.8 (1.4, 2.2)	1.8 (1.5, 2.2)	0 (− 0.6, 0.5)
≥ 1 Standard Alcoholic drinks per week, *n*/*N* (%)	32/77 (41.6)	20/78 (25.6)	15.92 (1.25, 30.58)^*	22/65 (33.8)	16/74 (21.6)	0.88 (0.68, 1.15)^+^
Mean number standard alcoholic drinks per week (95%CI)	4.6 (3.2, 6.1)	4.6 (2.8, 6.4)	0.1 (− 2.2, 2.3)	4.4 (2.8, 6)	6.2 (4.5, 8)	− 1.8 (− 4.2, 0.5)
Takeaway meals/week	1.9 (1.3, 2.5) (*n* = 77)	1.3 (0.7, 1.9)	0.6 (− 0.2, 1.4)	1.1 (0.8, 1.5) (*n* = 65)	1.2 (0.9, 1.5) (*n* = 74)	− 0.1 (− 0.5, 0.4)

^Percent difference (95% confidence interval), ^+^adjusted relative risk (95% confidence interval), ^#^standard deviation, ^$^metabolic equivalents, ^&^body mass index

*p < 0.05; **p < 0.01

### Effectiveness of the intervention

Self-efficacy data were available for 138/158 (87.3%) randomised participants (66/78 [84.6%] EMPOWER-SMS; 72/78 [92.3%] control). There were no significant differences in self-efficacy at 6 months between EMPOWER-SMS and control groups (7.6 [95% CI 7.3, 7.9] and 7.6 [7.3, 7.9], respectively, Adjusted mean difference 0 [95% CI − 0.4, 0.4], *p =* 0.924). A sensitivity analysis using complete case analysis was conducted to confirm the results, as there was more than 5% missing (completely-at-random) data and revealed no significant differences between EMPOWER-SMS and control groups (7.6 [95% CI 7.3, 7.9] and 7.6 [95% CI 7.3, 7.9], respectively, *p =* 0.925). Overall, few participants missed ≥ 1 doses of endocrine therapy medication. However, there was a significant difference between groups, with EMPOWER-SMS participants missing less doses than control (I: 3/42 [7.1%], C: 8/47 [17.0%], Adjusted RR 0.13 [95% CI 0.02, 0.91], *p* = 0.040). There were no other significant differences between groups (Table 2; Supplementary Material 3). The proportion of participants meeting guideline recommendations was low and there were no significant differences between groups (Supplementary Material 2). Only 5/78 (6.4%) participants in the EMPOWER-SMS group and 3/78 (3.8%) in the control group were ‘current smokers’ at follow-up.

Accelerometer data were available for 26/32 (81.2%) participants at baseline (14/16; 87.5% EMPOWER-SMS, 12/16; 75.0% control) and 20/32 (62.5%) participants at follow-up (10/16; 62.5% EMPOWER-SMS, 10/16; 62.5% control). Overall, self-reported physical activity was over-estimated compared to accelerometer data at follow-up with a very weak negative correlation (median GPAQ MET minutes/day [IQR] 300.00 [220.0, 1080.0], median accelerometer MET minutes/day [IQR] 168.39 [124.0, 227.1], *r = −* 0.09, *p =* 0.691) and was over-estimated in the EMPOWER-SMS group (strong negative, but not significant, correlation 240.00 [220.0, 1080.0] vs. 187.02 [146.5, 227.2]. *r* = *−* 0.61, *p* = 0.062) and control group (weak positive correlation 480.00 [120.0, 1650.0] vs. 164.8 [101.5, 201.4], *r* = 0.36, *p* = 0.307). Wilcox rank sum test found that accelerometer METS did not differ between groups (median [IQI] 187 [146.5, 227.2] and 164.8 [101.5, 201.4], respectively, *p* = 0.796).

At follow-up, there were no significant differences between groups for participants’ illness perceptions across the 8 domains (BIPQ; Supplementary Material 4). Overall, participants reported that breast cancer minimally *affected their life* (mean ± SD 4.3 ± 3.0) and did not *experience breast cancer symptoms much* (3.8 ± 2.8). Participants felt that their *treatment could help their breast cancer* (7.8 ± 2.3) and they felt that they *understand their breast cancer well* (8.1 ± 1.7). However, they felt they had moderate *control over their breast cancer* (5.0 ± 3.0), *concerns about their breast cancer* (5.7 ± 2.9) and reported that their breast cancer *affected them emotionally* (5.0 ± 3.0). When participants were asked what they thought caused their breast cancer (free-text), several common themes emerged: ‘stress’, ‘genetics/family history’, ‘not sure’, ‘bad luck/chance’, ‘age’, ‘oestrogen/hormones’ and ‘unhealthy lifestyle’ (included subthemes *unhealthy diet*, *physical inactivity*, *obesity*, *smoking* and *alcohol*). Participants ranked the three most important causes of their breast cancer as (1) unhealthy lifestyle, (2) stress and (3) not sure.

### Process evaluation

#### Evaluation of program delivery

A total of 8061 text messages were sent; 7925/8061 (98.3%) delivered successfully and 136/8061 (1.7%) bounced. Despite being instructed not to reply, participants replied 130 times (median per participant = 1; range = 1–80; outlier participant ID53 *n* = 80; next highest *n* = 17). She reported that ‘it’s nice to reply, so you get feedback’ (age 58). The most common replies were giving thanks, comments about personal health or complimenting EMPOWER-SMS (Box 1). One photo and 12 ‘reactions’ (2 ‘like’, 10 ‘loved’; ID160, age 40) were received. Thirteen messages included an emoji; usually a smiley face or heart. The most common themes that triggered a reply were self-care or managing side effects (30/53;57%), practical health tips (27/53; 50%), exercise (9/53; 17%) or diet (9/53; 17%). 
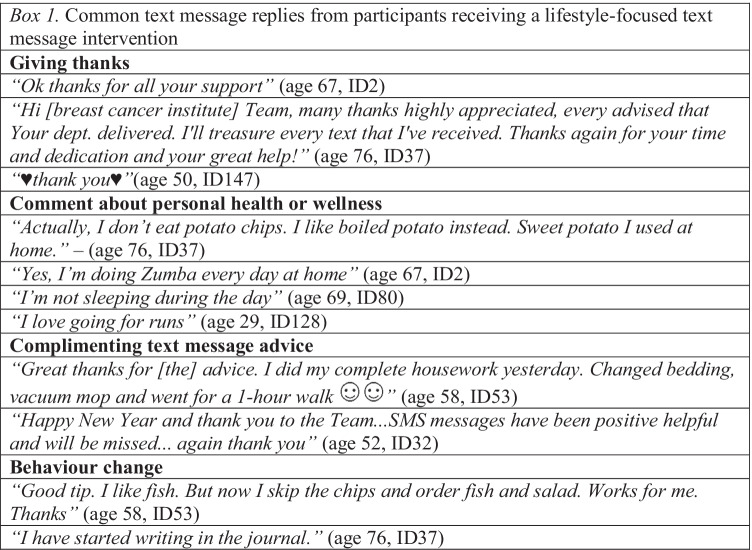


#### Program delivery costs

The cost to send one text message was $0.07USD and access automated delivery software for 20 months was $1523USD. Therefore, delivering 106 text messages to 76 EMPOWER-SMS participants and 2 text messages to 78 control participants cost $2097USD or $13.62USD per participant. Staff time to monitor incoming messages was estimated at 30 min/week.

#### Program acceptability and utility

Most (64/78; 82%) EMPOWER-SMS participants completed the intervention feedback survey (Table 3). The majority (57/64; 89%) read 75–100% of the messages and *agreed* or *strongly agreed* that the messages were easy to understand (64/64; 100%), useful (58/64; 90%) and motivated them to change their lifestyle (43/64; 67%). Half reported messages helped remind them to take their endocrine therapy medication (22/46; 48%). Most (51/64; 80%) participants thought that the six-month program length was *just right* and 10/64 (14%) it was *too short* or *much too short*. Most participants (46/64, 72%) saved the messages. However, only 28/64 (44%) shared them with family/friends and 7/64 (11%) forwarded the messages to others (Table 3), because it felt like ‘a personal experience’ [29].

**Table 3:** Intervention participants’ perceived acceptability and usefulness of the EMPOWER-SMS intervention

Characteristic	No./Total (%)^a^
Usefulness and understanding^b^	
Found messages useful	58/64 (90)
Majority of messages were easy to understand	64/64 (100)
Influence on motivation and behaviour change^b^	
Messages motivated lifestyle change	43/64 (67)
I increased by physical activity levels because of the messages	33/64 (52)
Messages helped remind me to take my medicines^c^	22/46 (48)
Message saving and sharing^d^	
Saved messages	46/64 (72)
Showed messages to family or friends	28/64 (44)
Forwarded messages to family or friends	7/64 (11)
Acceptability of program and message content	
Read 75-100% of messages	57/64 (89)
Number of messages per week was appropriate or ‘just right’^e^	57/63 (90)
Language of the messages was appropriate or ‘just right’^f^	56/62 (90)
6-month program was appropriate or ‘just right’^e^	51/64 (80)
6-month program was ‘too short’ or ‘much too short’^e^	10/64 (14)
Time of day receiving messages (9 am, 12 pm, 3 pm or 6 pm) was appropriate^b^	51/64 (80)

^a^Response rate was 64/78 (82%) of the intervention participants

^b^Response options were ‘strongly disagree, disagree, neutral, agree, strongly agree’. Reported the proportion that agree and strongly agree.

^c^Responses from participants taking endocrine therapy tablets.

^d^Responses were Yes or No. Reported proportion of participants who responded ‘Yes’

^e^Responses were Much too few/short, too few/short, just right, too many/long, much too many/long

^f^Responses were Too casual, casual, just right, formal, too formal

## Discussion

Accessible health education and support after breast cancer treatment remains a challenge in survivorship care [8, 30]. Despite participants’ perceptions that unhealthy lifestyle caused their breast cancer, this study highlighted that few participants were meeting secondary prevention guidelines; most participants had overweight or obesity, high body fat percentage, poor nutrition behaviours and poor mental health (anxiety, depression, stress), supporting a strong need for wide-reaching health support strategies. The EMPOWER-SMS RCT implemented a low-cost strategy to support self-efficacy, QOL and health outcomes via semi-personalised text messages for 6 months. Although EMPOWER-SMS was delivered as planned, it did not improve the primary outcome (self-efficacy). Adherence to endocrine therapy medication was high, but there was a significant difference between groups, favouring EMPOWER-SMS. Moreover, most participants rated EMPOWER-SMS easy-to-understand (100%), useful (91%), motivating (67%). Importantly, many participants felt the program duration was appropriate or wanted it to continue.

In terms of the primary outcome (self-efficacy), it is possible that the chosen scale (self-efficacy for managing chronic disease) was too broad to identify a change in this population. This scale combines self-rated self-efficacy across multiple domains including managing fatigue, pain, emotional distress and ‘things other than medications’. A previous study with cancer survivors found that self-efficacy can vary greatly across domains [18], indicating that evaluating them together may dilute their individual interpretation. Domain-specific baseline scores ranged from mean ± SD of 5.83 ± 2.56 for fatigue self-efficacy to 6.84 ± 2.23 for ‘things other than medications’ [18]. Comparatively, the current study’s baseline self-efficacy scores were high. A recent systematic review with meta-analyses of non-clinical populations found that digital health interventions had a small but positive impact on domain-specific self-efficacy for smoking but not healthy eating or physical activity [31] and only two were text message interventions (both not significant). Therefore, a domain-specific self-efficacy scale that aligned with EMPOWER-SMS content (physical activity, nutrition, fatigue, medication adherence) may have been more appropriate.

Despite the positive data relating to usefulness and motivation, it is also possible that text messages alone are insufficient to produce a change in self-efficacy for female breast cancer survivors. A systematic review with meta-analysis of healthy adults found that the effect of digital health interventions on self-efficacy decreased as the number of women included in the study increased [32]. Moreover, cancer survivors’ self-efficacy can be impacted by high levels of pain, depression and negative perceptions of cancer [18]. As there are no known similar text message studies targeting breast cancer survivors’ self-efficacy, direct comparison with previous research is not possible. However, a recent study with women of reproductive age found that text messages improved knowledge of breast cancer and breast self-examination but not self-efficacy for breast self-examination [33]. A recent systematic review with meta-analyses found that some eHealth interventions, namely interactive websites, improved breast cancer survivors’ self-efficacy [15], whereas another did not [34]. Further large-scale trials are needed to elucidate the relationship between text message interventions and domain-specific self-efficacy for breast cancer survivors.

The current study provided preliminary evidence that a lifestyle-focused text message intervention can improve endocrine therapy medication adherence compared to usual care. This result mirrors findings of increased medication adherence compared to control for patients with coronary heart disease [35], HIV positive youth taking anti-retroviral medication among [36], patients taking anti-diabetes oral tablets and beta-blockers [37] and asthma treatment [38]. One systematic review of 2742 patients with chronic diseases found that text message reminders *doubled* medication adherence compared to usual care [39]. Contrary to the EMPOWER-SMS intervention, which sent one, one-way (no replies) medication-related message per week, most previous interventions delivered *daily* text message reminders, and some required a response (two-way communication) [35–39]. A systematic review found 33–50% of women are non-adherent to their endocrine therapy tablets largely due to side effects (e.g. hot flushes, joint pain), but receiving social support and good clinician-patient communication can increase adherence [40]. Moreover, a pre-post study of 100 breast cancer survivors found evidence that daily medication reminder text messages with option to report side effects to a health professional was helpful for identifying adherence barriers and convenient for patients [41]. Qualitative results (focus groups, text message replies, free-text feedback survey responses) of the EMPOWER-SMS RCT found that the messages helped women feel supported and connected to their medical team between clinic visits [29]. To our knowledge, EMPOWER-SMS is the first lifestyle-focused text message intervention with only one medication-related message per week and found evidence of improved adherence to endocrine therapy medication. Since overestimation of self-reported adherence to endocrine therapy tablets compared to objective measures is common [42] and may limit study findings, further robust research is needed with a larger sample size of patients taking endocrine therapy medication to understand the full clinical potential of this resource-light intervention.

This study’s results are confounded by the global COVID-19 pandemic. In March 2020, COVID-19 restrictions in Sydney Australia included a stay-at-home order, which mandated working from home and closure of non-essential services [43]. For breast cancer survivors, telehealth replaced in-person clinic visits to lower chances of disease transmission [44]. This period was extremely stressful [45]. Pre-pandemic research estimated the incidence of anxiety and depression among breast cancer patients to be 18–33% [46] and 9–66% [46, 47], respectively and symptoms consistently improve across the first 2 years post-surgery [48]. In contrast, this study found that anxiety and depressive symptoms were above healthy ranges for 90/133 (68%) and 78/133 (58.6%) participants who were 1–2 years post-surgery. A systematic review of RCTs, including 764 adults diagnosed with depression, found that interventions that delivered 1–5 daily text messages were effective for improving depressive symptoms [49]. The current study, on the other hand, did not specifically recruit patients with depression or anxiety and message content targeted general lifestyle support, rather than daily support for depressive or anxiety symptoms. Despite this, this study found a text message intervention was feasible, low-cost, and acceptable for delivering health information and support to breast cancer survivors, even during COVID19 lockdowns.

Although the study had many strengths, including successfully testing a new co-designed digital health intervention in a high quality RCT with a population of culturally and ethnically diverse breast cancer survivors, several limitations should also be considered. Due to COVID-19 restrictions, half of the participants were followed-up by phone. Body composition data (body fat percentage, muscle mass percentage) were therefore unavailable and weight and waist-circumference measurements were self-reported, which limited power and accuracy [50]. Also, participants’ were a mean of 8 months post-treatment. Research suggests that continuity of patient care during and after treatment is important [30]. It is possible that earlier implementation would have been more beneficial to assist in the acute phase of transitioning from active treatment to independent health self-management. Despite this, participants found EMPOWER-SMS acceptable, useful and motivating for behaviour change and some wanted it to continue beyond 6 months. Future studies should consider beginning digital health survivorship support closer to completion of active treatment and include longer-term follow-ups.

## Conclusion

A semi-personalised lifestyle-focused text message intervention for women post-active breast cancer treatment was not associated with improvements in the primary (self-efficacy) or secondary outcomes but did have a small significant improvement on endocrine therapy medication adherence. Moreover, the program was deemed useful, acceptable, and motivating for behaviour change and medication adherence from a socioeconomically diverse population. The program provides a feasible, inexpensive, and easily scalable strategy for providing post-treatment health information and support remotely, including during COVID-19 lockdowns. The program could benefit from implementation closer to the end of active treatment and longer-term follow-up.

### Supplementary Information

ESM 1 (DOC 91 kb)

ESM 2 (DOCX 20 kb)

ESM 3 (DOCX 20 kb)

ESM 4 (RTF 101 kb)

## Data Availability

The datasets generated during and/or analysed during the current study are available from the corresponding author on reasonable request.
