# Supplementary material for: Supporting women’s health outcomes after breast cancer treatment comparing a text message intervention to usual care: the EMPOWER-SMS randomised clinical trial
Source: J Cancer Surviv. 2022 Apr 23;17(6):1533–45. doi: 10.1007/s11764-022-01209-9 (PMC9034445; doi:10.1007/s11764-022-01209-9)
Supplement: Supplementary file 2 — (DOCX 20 kb) [file 11764_2022_1209_MOESM2_ESM.docx]

*Supplementary Material 2.* Proportion of participants (n/N, %) meeting secondary prevention guidelines between the intervention (EMPOWER-SMS) and control groups at baseline and 6-month follow-up

|  | **Baseline** |  |  | **Six-month follow-up** | |  |
| --- | --- | --- | --- | --- | --- | --- |
|  | **EMPOWER-SMS**  **(n=78)** | **Control**  **(n=78)** |  | **EMPOWER-SMS**  **(n=78)** | **Control**  **(n=78)** |  |
| **Secondary prevention guidelines** | **No./Total (%)** | **No./Total (%)** | **Percent difference (95% CI)** | **No./Total (%)** | **No./Total (%)** | **Adjusted relative risk (95% CI)** |
| Physical activity, ≥600 METS^$^/week | 48/78 (61.5) | 44/78 (56.4) | 5.13 (-10.29, 20.54) | 47/68 (69.1) | 45/74 (60.8) | 1.14 (0.89, 1.45) |
| BMI^^^ <25kg/m^2^ | 19/78 (24.4) | 23/77 (29.9) | 5.51 (-8.46, 19.48) | 17/60 (28.3) | 22/73 (30.1) | 1.02 (0.95, 1.09) |
| Waist circumference ≤80cm | 8/78 (10.3) | 20/76 (26.3) | 16.06 (4.09, 28.03)^**^ | 7/56 (12.5) | 15/68 (22.1) | 0.98 (0.91, 1.05) |
| Fat Mass Percentage ≤32% | 2/74 (2.7) | 8/71 (11.3) | 8.56 (0.33, 16.8) | 2/25 (8) | 3/27 (11.1) | - |
| Skeletal muscle mass percentage, ≥24.2 | 74/74 (100) | 70/70 (100) |  | 25/25 (100) | 27/27 (100) | - |
| ≥14 servings of fruit/week | 27/77 (35.1) | 33/78 (42.3) | -7.24 (-22.53, 8.05) | 16/65 (24.6) | 28/74 (37.8) | 0.66 (0.41, 1.05) |
| ≥35 servings of vegetables/week | 10/77 (13) | 7/78 (9) | 4.01 (-5.82, 13.84) | 10/65 (15.4) | 9/74 (12.2) | 1.14 (0.51, 2.55) |
| ≤2 servings of red meat/week | 40/77 (51.9) | 50/78 (64.1) | -12.15 (-27.58, 3.27) | 46/65 (70.8) | 52/74 (70.3) | 1.03 (0.87, 1.21) |
| ≥2 servings of fish/week | 34/77 (44.2) | 35/78 (44.9) | -0.72 (-16.36, 14.93) | 29/65 (44.6) | 34/74 (45.9) | 0.94 (0.7, 1.25) |
| ≤10 standard alcoholic drinks/week | 74/76 (97.4) | 76/78 (97.4) | -0.07 (-5.09, 4.96) | 63/65 (96.9) | 72/74 (97.3) | -0.37 (-5.97, 5.22) |
| **Mental health within ‘normal’ range** |  |  |  |  |  |  |
| Depressive symptom score ≤9 | 25/73 (34.2) | 26/77 (33.8) | -0.48 (-15.65, 14.69) | 23/63 (36.5) | 32/70 (45.7) | 1.21 (0.98, 1.49) |
| Anxiety symptom score ≤7 | 25/73 (34.2) | 26/76 (34.2) | -0.04 (-15.28, 15.2) | 17/63 (27) | 26/70 (37.1) | 1.13 (0.96, 1.33) |
| Stress score ≤14 | 45/74 (60.8) | 49/77 (63.6) | 2.83 (-12.64, 18.29) | 43/63 (68.3) | 49/70 (70) | - 1. (0.73, 1.52) |
| Too few cases to conduct adjusted relative risk ^$^Metabolic Equivalents ^^^Body Mass Index ^**^p<0.01 | | | | | | |
