# Supplementary material for: Supporting women’s health outcomes after breast cancer treatment comparing a text message intervention to usual care: the EMPOWER-SMS randomised clinical trial
Source: J Cancer Surviv. 2022 Apr 23;17(6):1533–45. doi: 10.1007/s11764-022-01209-9 (PMC9034445; doi:10.1007/s11764-022-01209-9)
Supplement: Supplementary file 3 — (DOCX 20 kb) [file 11764_2022_1209_MOESM3_ESM.docx]

|  | **Baseline** |  |  | **Six-month follow-up** | |  |
| --- | --- | --- | --- | --- | --- | --- |
|  | **EMPOWER-SMS**  **(n=78)** | **Control**  **(n=78)** |  | **EMPOWER-SMS**  **(n=78)** | **Control**  **(n=78)** |  |
|  | **Mean (95%CI)** | **Mean (95%CI)** | **Mean difference (95%CI)** | **Adjusted mean (95%CI)** | **Adjusted mean (95%CI)** | **Adjusted mean difference (95%CI)** |
| Physical functioning | 81·4 (77·3, 85·5) | 79·7 (75·6, 83·7) | 1·7 (-4·1, 7·6) | 83·8 (81, 86·6) | 81·1 (78·5, 83·8) | 2·7 (-1·2, 6·5) |
| Role functioning | 77·2 (71·3, 83·1) | 81·4 (75·6, 87·2) | -4·2 (-12·5, 4·1) | 84·1 (79·4, 88·8) | 84 (79·7, 88·4) | 0·1 (-6·3, 6·5) |
| Emotional functioning | 67 (61·1, 72·9) | 71·5 (65·6, 77·3) | -4·5 (-12·8, 3·8) | 71·3 (66·8, 75·8) | 70·7 (66·4, 75) | 0·6 (-5·6, 6·8) |
| Cognitive functioning | 73·5 (67·3, 79·7) | 73·5 (67·4, 79·6) | 0 (-8·8, 8·7) | 76 (72·2, 79·8) | 74 (70·4, 77·6) | 2 (-3·3, 7·3) |
| Social functioning | 78·3 (71·9, 84·7) | 76·7 (70·4, 83) | 1·6 (-7·4, 10·6) | 76·2 (71, 81·5) | 80·5 (75·5, 85·4) | -4·3 (-11·5, 3) |
| Fatigue | 43·7 (38, 49·4) | 41·9 (36·3, 47·5) | 1·8 (-6·2, 9·8) | 39 (34·3, 43·8) | 39·7 (35·2, 44·2) | -0·6 (-7·2, 5·9) |
| Nausea and vomiting | 8·8 (5·4, 12·2) | 5·8 (2·4, 9·1) | 3 (-1·8, 7·8) | 7·6 (4·7, 10·6) | 6·1 (3·4, 8·9) | 1·5 (-2·5, 5·5) |
| Pain | 30·9 (24·8, 37·1) | 25·4 (19·4, 31·5) | 5·5 (-3·1, 14·1) | 29·1 (24, 34·3) | 27·5 (22·6, 32·3) | 1·7 (-5·4, 8·7) |
| Dyspnoea | 14·7 (9·4, 19·9) | 19·9 (14·7, 25·1) | -5·2 (-12·6, 2·1) | 17·5 (13·3, 21·7) | 13·8 (9·8, 17·8) | 3·7 (-2·1, 9·6) |
| Insomnia | 45·6 (37·7, 53·5) | 41·9 (34·1, 49·7) | 3·7 (-7·4, 14·8) | 45·3 (39·1, 51·6) | 43·8 (37·9, 49·7) | 1·5 (-7·1, 10·1) |
| Appetite loss | 18·9 (13·3, 24·4) | 11·1 (5·6, 16·6) | 7·7 (0, 15·5) | 12·7 (8·1, 17·2) | 9 (4·7, 13·3) | 3·7 (-2·6, 10) |
| Constipation | 18·4 (12, 24·8) | 18·4 (12, 24·7) | 0 (-9, 9·1) | 15·5 (10·7, 20·4) | 19·8 (15·2, 24·3) | -4·2 (-10·9, 2·5) |
| Diarrhoea | 10·5 (6·4, 14·6) | 5·1 (1·1, 9·2) | 5·4 (-0·4, 11·1) | 12·3 (7·4, 17·1) | 7·9 (3·3, 12·5) | 4·4 (-2·3, 11·1) |
| Financial difficulties | 25·4 (17·8, 33) | 20·5 (13, 28) | 4·9 (-5·8, 15·6) | 22·2 (17·4, 26·9) | 22·6 (18·1, 27·1) | -0·4 (-7, 6·1) |
| Body image | 66 (59·9, 72) | 74·5 (68·5, 80·4) | -8·5 (-17, -0·1)* | 73·8 (69·8, 77·7) | 74·4 (70·7, 78·1) | -0·6 (-6, 4·8) |
| Sexual function | 22·2 (16·9, 27·5) | 15·8 (10·6, 20·9) | 6·4 (-1, 13·8) | 21·8 (17·8, 25·8) | 20·8 (17·1, 24·4) | 1 (-4·5, 6·5) |
| Sexual enjoyment | 60·6 (51·7, 69·5) (n=33) | 39·1 (29·6, 48·5) (n=29) | 21·5 (8·6, 34·5)** | 56·2 (45·8, 66·6) (n=35) | 51·8 (39·1, 64·5) (n=30) | 4·4 (-12·7, 21·5) |
| Future perspective | 46·2 (38·2, 54·2) | 49·4 (41·4, 57·3) | -3·1 (-14·4, 8·1) | 50·3 (44·3, 56·3) | 52·8 (47·2, 58·4) | -2·5 (-10·7, 5·7) |
| Systemic therapy side effects | 24·9 (20·6, 29·3) | 23·9 (19·6, 28·2) | 1 (-5, 7·1) | 22·4 (19·7, 25·1) | 22·8 (20·2, 25·3) | -0·4 (-4·1, 3·4) |
| Breast symptoms | 27·5 (22·3, 32·7) | 21·9 (16·7, 27) | 5·6 (-1·7, 12·9) | 22 (18·3, 25·8) | 22·8 (19·2, 26·3) | -0·7 (-5·9, 4·4) |
| Arm symptoms | 25·3 (19·9, 30·8) | 23·2 (17·8, 28·6) | 2·1 (-5·6, 9·8) | 20·2 (15·7, 24·7) | 22·5 (18·2, 26·7) | -2·3 (-8·5, 3·9) |
| Upset by hair loss | 52·4 (36·1, 68·7) (n=21) | 44 (29, 59) (n=25) | 8·4 (-13·8, 30·5) | 45·8 (27·5, 64) (n=18) | 48·8 (34·1, 63·5) (n=29) | -3·1 (-26·6, 20·5) |
| *p<0.05 **p<0.01 |  |  |  |  |  |  |

*Supplementary material 3*. Quality of life subscales at baseline and six-month follow-up between groups (EMPOWER-SMS vs control) measured by the European Organization for Research and Treatment of Cancer Quality of Life Questionnaire–Core and Breast Cancer sub-scales
