# Supplementary material for: Supporting women’s health outcomes after breast cancer treatment comparing a text message intervention to usual care: the EMPOWER-SMS randomised clinical trial
Source: J Cancer Surviv. 2022 Apr 23;17(6):1533–45. doi: 10.1007/s11764-022-01209-9 (PMC9034445; doi:10.1007/s11764-022-01209-9)
Supplement: Supplementary file 4 — (RTF 101 kb) [file 11764_2022_1209_MOESM4_ESM.rtf]

Supplementary Material 4. Brief illness perceptions questionnaire responses (by group allocation) and proportion of themes when asked 'what are the three most important causes of my illness'&
	
	Baseline			Six-month follow-up		
	EMPOWER-SMS
(n=78)	Control
(n=78)		EMPOWER-SMS
(n=78)	Control
(n=78)		
	Mean (SD+)	Mean (SD+)	Mean difference (95%CI#)	Mean (SD+)	Mean (SD+)	Adjusted Mean difference (95%CI)	
How much does illness affect your life?	4·8 (3·1)	4 (3·1)	0.9 (-0.2, 1.9)	4·4 (3)	4·2 (3·1)	-0.1 (-0.8, 0.7)	
How long do you think your illness will continue?	4·6 (3·4)	3·8 (3·4)	0.8 (-0.3, 1.9)	4·2 (3·2)	4·1 (3·5)	0.1 (-1.1, 1.3)	
How much control do you feel you have over your illness?	4·9 (3·3)	5·7 (3)	-0.7 (-1.8, 0.3)	4·7 (3)	5·4 (3)	-0.4 (-1.4, 0.5)	
How much do you think your treatment can help your illness?	8·5 (1·7)	8·1 (2)	0.4 (-0.2, 1)	7·9 (2·1)	7·8 (2·5)	-0.1 (-0.9, 0.7)	
How much do you experience symptoms from your illness?	4 (3·1)	4 (3)	-0.1 (-0.9, 0.7)	3·6 (2·9)	3·9 (2·7)	-0.1 (-0.8, 0.6)	
How concerned are you about your illness?	6·6 (3)	5·5 (3·3)	1 (0, 2)	6·1 (2·7)	5·3 (3·1)	0.4 (-0.4, 1.2)	
How well do you feel you understand your illness?	8 (2)	7·9 (2·1)	0 (-0.6, 0.7)	7·9 (1·6)	8·3 (1·7)	-0.4 (-1, 0.2)	
How much does your illness affect you emotionally?	5·8 (3·3)	4·8 (3·3)	1 (-0.1, 2)	5·4 (2·9)	4·6 (3·1)	0.4 (-0.4, 1.1)	
	# Theme/Total responses (%)				
	n=64	n=70	N=132	n=62	n=68	N=130	
Most important cause of my illness	Unhealthy lifestyle 35/146 (24·0)	Unhealthy lifestyle 28/166 (16·9)	Unhealth lifestyle 63/312 (20·2)	Unhealthy lifestyle 29/172 (40·3)	Stress 35/192 (18·2)	Unhealthy lifestyle 56/364 (15·4)	
Second most important cause of my illness	Stress 25/146 (17·2)	Stress 22/166 (13·2)	Stress 47/312 (15·1)	Not sure 23/172 (13·3)	Unhealthy lifestyle 27/192 (13·7)	Stress 55/364 (15·1)	
Third most important cause of my illness	Genetics 13/146 (8·9)	Not sure 20/166 (12·0)	Not sure 32/312 (10·2)	Stress 20/172 (11·6)	Not sure 21/192 (10·9)	Not sure 44/364 (12·1)	

&Brief illness perceptions questionnaire responses (mean score out of 10) and proportion of themes (# theme/total responses)
+Standard Deviation
#Confidence Interval
